# Book Reviews

**Published:** 1818-05

**Authors:** 


### CRITICAL ANALYSIS

### *OF RECENT PUBLICATIONS,*

IN THE

### DIFFERENT BRANCHES OF PHYSIC, SURGERY, &c.

[This department has for some numbers past been so entirely occupied by matters of a temporary interest, as to make us much in arrear with the Transactions of Societies, and other periodical works.]

*Philosophical Transactions of the Royal Society of London, for the Year* 1817. Part II. London: Nicol, Pall Mall.

*On the Passage of the Ovum from the Ovarium to the Uterus in Women;* by Sir EVERARD HOME, Bart. V.P.R.S.

"NO subject (says Sir Everard) connected with physiology has more employed the attention of the anatomist and philosopher than the first formation of the embryo in the class mammalia; and yet, even at this day, when the same subject has been completely investigated in oviparous animals, and it is known that an ovum is formed in the ovarium of the quadruped, the circumstances respecting its impregnation have not been ascertained.

"The great Harvey, although supplied by the munificence of his king with deer in all the different stages, after being fit for the male, was unsuccessful. John Hunter, who prosecuted the same enquiry in the ewe, also failed. His brother, Dr. William Hunter, in his splendid work on the Gravid Uterus, has given the most correct representations of the human embryo, from the end of the third week till the time of birth, but has not said any thing upon the subject of impregnation.

"Haighton and Cruikshank, by experiments on rabbits, confirmed the opinion of De Graaf,* that an ovum is carried from the ovarium into the uterus, but by mistaking the corpus luteum for the effect of impregnation, instead of the substance in which the ovum is formed, which, at that time, was the generally received opinion, got entangled in theoretical opinions, which misled them in their further enquiries.

"In this state of our knowledge upon this most interesting subject, accident has done what no predetermined experiments had accomplished: it has enabled me to detect the ovum in the human uterus. It is so small, that, had not the uterus been previously hardened in spirit as well as the ovum itself, it probably would have escaped observation; and, after it was found, it could not

"* De Graaf's Observations are mentioned in the 7th vol. of the Philosophical Transactions, p. 4052. In the same volume, p. 4018, Dr. Kerkringius's Observations concerning Eggs to be found in all sorts of Females are noticed."

have been identified to be the ovum from which a child was to be produced, had it not been brought under the eye of Mr. Bauer, the only person, I may say, in this or any other country, who could so correctly apply to it the powers of the microscope, as to determine its form; could so separate its parts on the field of the microscope, as to display its organization; and so delineate what he saw, as to convey distinct notions that it was the first rudiments of a child."

We conceived that we had met with optical deception enough in Sir Everard Home's early papers on vision, in which he demonstrated so accurately what Mr. Hunter was conscious he had left in too imperfect a state for publication, and what Sir Everard afterwards found was illusive. Yet this was demonstration, and with the naked eye.—Now we are called upon to admit a discovery in a subject first hardened by spirits, and, in this altered condition, submitted to the microscope of one who alone is capable of detecting what no one else could detect, and, we suppose, no one else delineate.—But, let us return to the history. A servant-maid died epileptic,—

"After death, the uterus showed signs of pregnancy, and, from the statement that has been given, she appears to have been impregnated on the 7th of January, eight days before her death; for, although she was known to have a lover, there are circumstances to prove, that she could not have seen him after that time, nor for many days before.

"The uterus having been hardened in spirit, with the assistance of Mr. Clift, I examined the parts. The right ovarium had a small torn orifice upon the most prominent part of its external surface: we slit it open in a longitudinal direction, in a line close to the edge of this orifice: the orifice was found to lead to a cavity filled up with coagulated blood, and surrounded by a yellowish organized structure. Upon opening into the cavity of the uterus, its inner surface was covered with an exudation of coagulable lymph, beautifully represented in the drawing, (Pl. VIII.); the ovum lay concealed among the long fibres of coagulable lymph near the cervix, and was brought to view by separating them with the point of a needle which I employed in making the search. As soon as it was disentangled, it rose up, moving along with the loose ends of the fibres into the spirit, by which the parts were covered. It had an oval appearance: one portion of it was quite white, the other semi-transparent; but, soon after, being exposed to the spirit, the whole became opaque. The os tincæ was entirely shut up with a strong solid jelly, the two orifices at the angles of the uterus, by which it communicates with the fallopian tubes, were both pervious.

"As the ovum was so extremely small as to admit of dispute, whether it was one or not, I carried it immediately to Kew, to Mr. Bauer, who, after examining it, said that it looked like the egg of an insect. His drawings of the ovum and uterus show to what an excellence microscopical observations can be carried, since, in so small a particle of animal matter, he has pointed out the effects of impregnation even before any part of the vascular system had been formed, and where only the two projecting points within the ovum had been marked out as the future situations of the heart and brain. These two points are still to be distinguished in the ovum in a dried state, and that towards the broadest end is the largest.

"Small as this ovum is, it bears a very fair proportion to that represented by Dr. Hunter at the end of three weeks; and, had this woman lived twenty-four hours longer, the ovum would probably have, in that time, been united to the fibrous structure surrounding it, and appeared secluded from the cavity of the uterus in the same degree as Dr. Hunter's is represented to be."

As this engraving is not coloured, the parts so beautifully seen, to say the least, are not more conspicuous, nor more beautifully exhibited, than those muscles of the eye to which we before alluded. The uncertainty of microscopical observations with large magnifiers is universally admitted. Perhaps, the mode of using these instruments may be improved since Mr. Hunter's time; but, if the art is confined to a single person, the result will, we fear, be attended with some scepticism.* The following is Mr. Bauer's description.

"'On closely examining the subject under the microscope, I found it consisted of membrane, which, considering the extreme

---

* We shall take the liberty, on this occasion, of transcribing Mr. Hunter's opinion of microscopical evidence:—

"I am led to believe that we may be deceived by the appearances viewed through a magnifying glass; for, although objects, large enough to be seen by the naked eye, are the same when viewed through a magnifying glass which can only magnify in a small degree, yet, as the naked eye, when viewing an object rather too small for it, is not to be trusted, it is much less to be depended upon when viewing an object infinitely smaller, brought to the same magnitude by a glass. In such a situation, respecting our eye, all the relative objects by which the eye, from habit, judges with more nicety of the object itself, are cut off: the eye has likewise a power of varying its forms, adapting it to the different distances of the parts of an object within its compass, making the object always a whole; but a magnifying glass has no such power: for instance, in viewing a spherical body, a magnifying glass must be made to vary its position, and bring in succession the different parts of the hemisphere into so many focal points; every part separate not having the same relative effect on our organ of vision as minuteness of the subject, is of considerable thickness and consistence, very little transparent, quite smooth, and milk white,

---

when they are all seen at the same time; and the eye, under such circumstances, being unable to vary itself sufficiently to alter the focal distance of the glass, is the reason why rounded bodies appear of different shapes, giving the shape only of the part that is within the focus of the glass, placed upon an undefined plane; and if it should have more focal points than one, then there is an increase of parts; and this will vary according to the opacity or transparency of the body. It may also be remarked, that from habit our minds are informed by the necessary actions of our body: therefore, the eye taking on the necessary actions (as it were instinctively), adapting itself at once to the circumstances of the object, gives an intelligence to the mind independent of the real impression of the object, so that both the impression and the consequent action give information; but this cannot be effected by glasses,—for the different focal distances of the hemisphere do not accord with those to which we vary our eyes in adapting them to the distances of the different parts of a rounded body: we are, therefore, left to the impression alone, which is new, and consequently imperfect, the centre being too near for the circumference to be seen at one distance, and the circumference, when seen, bringing the centre within the focus, so as to obscure it: for an eye, with a given focus, which can vary it in a certain degree when viewing objects alone, yet, when looking through a magnifying glass of any power, must now vary the distance of the object according to the magnifying power in the glass, the eye not being able to vary the focal distance of both; and this, probably, in an inverse degree to the magnifying power of the glass. This may be observed to take place in very short-sighted people, for in them the eye has the least variation respecting distance. A rounded body may be just of such a size as shall have either of its parts out of the focal distance of the eye, and must be moved to and fro, alternately, before the centre and circumference can be seen; and, indeed, it is only having a spherical body, of a size proportional to the length of focus, to produce the same effect in every eye.

"The appearance in a transparent body, when viewed through a magnifying glass, are still more fallacious than of an opaque one; for an opaque body gives only the reflected light, which, however, will vary according as the rays come on the object. The moon, an opaque body, gives us various shapes, and therefore shews only the light and shade arising from the irregularity of the surface; but a semi-transparent body, like a red globule, gives both the reflection of the light from the surface, and also the refraction of other rays of light, which vary according to the direction of the light thrown upon the object respecting our eye.

"In some transparent bodies we have still a greater variety, for we have both the reflex and refracted light, and these vary accordforming a kind of bag or pouch of an irregular oval shape, not quite $\frac{19}{200}$ parts of an inch in length, and in its middle about $\frac{10}{200}$ parts of an inch broad; on one side, it has an elevated ridge, or large fold, along the whole length; and, on the opposite side, it is open nearly the whole length, but has no appearance of being torn, the edges of the membrane being smoothly rolled inwards, which gives it much the shape of a little shell of the genus Voluta.

" ' When laid on glass, the membrane admitted easily to be laid open on both sides, with the point of a fine camel's-hair pencil. When thus opened, I found it contained another smaller bag somewhat less than $\frac{13}{200}$ parts of an inch long, and not quite $\frac{5}{200}$ parts of an inch broad, ending at the upper extremity nearly in a point; but the under extremity was very obtuse or truncate, and in the middle it was slightly contracted, which gave it the appearance of a young seed-capsule of some plants that contain only two seed-kernels.

" ' This inner bag consisted of a seemingly very thin, perfectly smooth, and glossy membrane, which seemed to have considerable strength, as it bore to be rubbed pretty strongly, not only with the camel's-hair pencil, but also with the point of the quill; it seemed to be filled with some thick slimy substance, as an impression made on it with the point of the quill remained for a considerable time visible: it contained two round corpuscles, apparently more opaque, and of a yellowish tint; they were not only visible through the transparent membrane, but they swelled the membrane over them, so that the light and shade made them to be distinctly seen; and, by slightly pressing the bag with the quill between the two corpuscles, they could be separated to a greater distance from each other, but, on putting more moisture upon the subject, they returned quickly to their former position. This little bag was along its whole length, with its back part strongly fixed to the outer membrane,—at least, I could not move it with the camel's-hair pencil, and more force I was afraid to employ.

" ' I attempted to open the little bag, if possible, to extract the corpuscules; but, on piercing, with the point of a very small needle, the upper extremity, a thick slimy matter, like honey, came out, and with the membrane adhered to the needle, so that I could no farther proceed; and, fearful of spoiling the whole, I gave up the attempt, and left the subject on the glass to dry; but I observed, as the spirit and moisture gradually evaporated, so the

---

ing to the distance of the object from the eye, or the distance of the light.

" If the transparent body is not perfectly round, or is by any circumstance broken in the uniformity of structure on which transparency depends, (which, I conceive, happens to the red globules when diluted in the serum,) then the different reflections and refractions will give to the eye the impression of so many shapes."—*Hunter on the Blood*, p. 42,—Note.

little bag flattened, and, as if melting, shrunk into the outer membrane, and almost disappeared, but, in a strong light, was still visible in the microscope.

"'When quite dry, its colour changed to a light yellowish brown, and it lay quite loose on the glass, except at the upper extremity, where I attempted to open it: it was strongly glued to the glass, and it required several times to be moistened at that part with water, to remove it from the glass.

"'I have now placed it between two pieces of talc in an ivory slider; and, in a strong light, the two corpuscules may still be seen through a common magnifying glass.'"

We are sure both these gentlemen will wish to suspend their own decision till other experiments are made, which may be more readily done on such larger animals as may be constantly under our observation; and our experiments on which, being less doubtful, may admit of a greater certainty as to dates. Our country readers, in these respects, may have advantages which will much more than compensate for their want of such magnifiers.

Before we dismiss this paper, we wish to know, by what means a discovery was made, that this yellowish structure was *organized;* or, why Sir Everard should be so inattentive to that accuracy he once seemed to have learned from his master, as to call *coagulated* lymph *coagulable.* Was it still fluid ?—If not, how can that be coagulable which is already coagulated.

These are among the difficulties we meet with in this paper,—difficulties which the number and beautiful execution of the engravings did not remove.

*Some farther Observations on the Use of the Colchicum Autumnale in Gout;* by the same.

*The distinguishing Characters between the Ova of the Sepia, and those of the Vermes Testacea, that live in Water, explained;* by the same.

This paper is valuable as affording at least a scrap of those manuscript treasures, which have hitherto not found their way to the public in any form whatever.

"The following observations respecting land-snails were made in the year 1773, the first year that I was initiated in comparative anatomy, under Mr. Hunter. He kept snails to ascertain their mode of breeding; and the notes that were made at the time in my own hand-writing, I now copy.

"August 5, 1773.—A snail laid its eggs, and covered them over with earth; Mr. Hunter took one out and examined it; the egg was round, its covering strong, and of a white colour, with a degree of transparency; it had no yelk; a small speck was observable with a magnifying glass in the transparent contents.

"On the 9th, no apparent change had taken place. On the 11th, the speck had enlarged, but was too transparent to admit of its form being distinguished; upon moving the speck, it fell out of its place.

"On the 12th, the embryo was indistinctly seen.

"On the 15th, the embryo filled a quarter part of the egg, but the different parts were still indistinct.

"On the 18th, the body of the embryo had become larger, and the covering thicker.

"On the 19th, the coverings or shells of all the eggs were more or less dissolved, so much so, that Mr. Hunter thought all the eggs were rotting, and the whole brood of young would be lost.

"On the 20th, the young were hatched, and the shells completely formed.

"On the 23d, when the young snails were put in water, their bodies came out of the shell, as in full grown snails.

"On the 24th, they all deserted their nests."

*Some Account of the Nests of the Java Swallow, and of the Glands that secrete the Mucus of which they are composed;* by the same.

These nests are mentioned by all commercial as well as philosophic travellers, as making a considerable article of trade, and forming a most luxurious dish among the Chinese. Sir Stamford Raffles, by the same accurate observation which he turned to so good an account in whatever he noticed, observed, that the substance of which these nests was composed, was voided from the mouth of the bird, and not without strong and frequent attempts. Professor Brand analysed them, and found them to consist of a substance intermediate between gelatine and albumen. The bird is double the size of our common swallow, and is found to have a peculiar apparatus.

"The only difference between the glands of the migrating swallow, and those of the blackbird, is the smallness of the reservoir; the surface of the gullet upon which the external openings of the glands are seen is exactly the same; there is not, in the one or the other, any apparatus for secreting mucus, which is not common to birds in general.

"In the Java swallow, we have, on the other hand, a structure of a particular nature; there is a membranous tube surrounding the duct of each of the gastric glands, which, after projecting into the gullet for a little way, splits into separate portions like the petals of a flower: for what purpose so extraordinary an apparatus could be provided, would, probably, have puzzled the weak intellects of human beings, and many wild theories might have been formed respecting it, had not the animal matter of which the bird's nest is composed, and the accurate observation of Sir Stamford Raffles, who had no doubt that the materials of the nest were produced from the gullet, led to the discovery of its use.

"That the mucus of which the nest is composed, is secreted from the surface of these membranous tubes, there is no more doubt, than that the gastric juice is secreted from the glands whose ducts these tubes surround; and this confirms an opinion which I have adopted for many years, that membranes on which no glandular structure could be seen, were capable of secreting mucus; and now that we find those membranes, where their surfaces are so much magnified, exhibit no glandular structure, we may, without the chance of more accurate observers refuting us, be satisfied that no such structure exists."

We were not aware, that this conjecture concerning the impossibility of detecting glands in some mucous membranes was entirely new, nor can we perfectly reconcile this remark with the title of the paper.

Some interesting observations follow on the peculiarities by which this and several other animals are adapted for the country they inhabit.

*Observations on the Gastric Glands of the Human Stomach, and the Contraction which takes place in that Viscus.* By the same.

By means of Mr. Bauer's magnifiers, six 100th parts of a square inch were magnified 15 times in diameter, or 225 times in superficies. Another part was magnified 900 times in superficies. The drawings are extremely beautiful; and it appears that these larger magnifiers correct the errors of the common lens.

"The structure upon the upper arch of the stomach, which, when magnified by a common lens, had the appearance of glands, is shown by Mr. Bauer to be made up of cells in the form of a honeycomb, the sides of which are not formed by doublings of the membrane; for no stretching of the cells alters the form of their orifices, but are regular partitions constructed between the cells. This honeycomb structure consists of cells of the greatest depth in this particular situation, but it is met with over the whole surface of the cardiac portion of the stomach; only the appearance is so faint as to require a great magnifying power to render it visible. In the pyloric portion, the cells, in general, have the same appearance; but there are small clusters, the sides of which rise above the surface, giving the appearance of foliated membranes. In the duodenum, this takes place in a greater degree, and the loose edges of these membranes, when entangled in the mucus that covers them, puts on an appearance of rounded glandular bodies; but these admitted of being expanded so as to explain the deception.

"The description which I have given of the internal membrane of the stomach, proves how nearly my late ingenious friend, Dr. George Fordyce, who examined its surface with very inferior means to those employed upon the present occasion, had approached the truth, when he declared it to be composed of cellular membrane."

The similarity, however, in the appearance of the œsophageal part of the human stomach to that of birds, might fairly lead to a conclusion that the structure and office is similar.

In the Lectures delivered at the College, Sir Everard marked, in a striking manner, the extraordinary provision made by nature for the animals inhabiting the most unproductive parts of the globe. Nothing can be more interesting than these views of benevolence and design in the whole economy of nature. But we may have too much of a good thing; or, rather, a good thing may be hacknied.

"I have shown, (says the author,) upon more occasions than one, that the gastric glands are both largest and most numerous in those animals destined to inhabit the least fertile regions of the earth, and are smallest as well as fewest where the supply of food is most abundant, to prevent the body being injured by the effects of over-feeding. If this arrangement was necessary in animals, it became still more so in man, whose means of procuring and preparing food for himself so much exceed those of all other animals, and who is, contrary to his reason, too readily disposed to carry the indulgencies of the table to excess. In him the gastric glands, as it was natural to expect, are so small, as to require the aid of Mr. Bauer's microscope to prove that they belong to the same series of structures as the gastric glands of the ostrich, which admit of being minutely examined by the naked eye."

The benevolence of such a provision in unproductive districts is apparent; but we cannot so easily see the necessity or advantage of a contrary apparatus to prevent the effects of an animal over-eating himself. Least of all can we see the advantage of enabling man thus to indulge with impunity; and, if this provision be made in favour of gluttony, may not the bloated drunkard complain how much he has been overlooked. Nor, indeed, is such a provision of Nature apparent; for we have not yet learned that the human inhabitants scattered throughout Arabia Deserta have larger or more numerous gastric glands than those who are pampered n this luxurious metropolis.

### *Edinburgh Medical and Surgical Journal*, No. LIV. for April, 1818.

Art. I.—*Case of Gun-shot Wounds of the Heart.* By John H. Fuge, Esq. Member of the Royal Medical Society of Edinburgh.

Two very interesting cases are related of gun-shot wounds in the heart. The first, by Mr. Fuge, is by far the most valuable. The patient, from the time he was able to communicate his sensations till his death, was certain that the issue must be fatal: but the length of time he lived after so serious an injury is scarcely to be credited, but for the respectability of the author, and the accurate manner in which all the facts are related.

"I was informed, (says the ingenious relater,) by one of the grenadiers who assisted in conveying him from the field, that, on the recovery of his senses, he expressed his conviction that the wound, from the very peculiar sensations he felt, would prove mortal. The loss of blood appeared not to have been copious at the time he was taken from the ground.

"On the day subsequent to that of his death, the body was opened, in the presence of my assistants and some other professional gentlemen. On raising the sternum, the left side of the thorax was found to contain about two quarts of a serous fluid, slightly tinged with blood; the lung was reduced to a small solid mass, adhering strongly to the spine; the pleura costalis exhibited marks of high inflammatory action; the pericardium was thickened, and unusually distended: on cutting into it, about half a pint of the same coloured fluid as that found in the cavity of the pleura was collected. The heart, on being superficially viewed, was found to have partaken of the prevailing inflammation; a thin coat of coagulable lymph adhered to it, and near its apex was seen a small attachment of coagulated blood. On raising the heart from the pericardium, I was struck with the appearance of a transverse opening, about one inch in length, penetrating the right ventricle, near the origin of the pulmonary artery. On removing the heart, by cutting through the great blood-vessels, the ball was sought for, and taken up from the cavity of the pericardium. By tracing its course, it became evident that it must have remained in the right auricle, as the tricuspid valve had a circular lacerated opening in it, near its attachment to the muscular structure of the ventricle. The contents of the right side of the thorax were unaffected by inflammation.

"In this very extraordinary case, life was prolonged for nearly fourteen days; and, if the usual antiphlogistic means had been resorted to at the time when high inflammatory action prevailed, it might, in all probability, have been extended to a much later period.

"Numerous instances of wounds of the heart are related by Senac, Morgagni, and others; but in those it does not appear that the perforations were so complete as in the present case. It is by no means difficult to account for the prolongation of life, where a sword, or any other sharp instrument, have been the weapons used; but how to explain the circumstance of the non-effusion of blood into the pericardium, at the moment of the accident, as well as subsequently, is a difficulty which hitherto I have been unable to solve."

We are less surprised than the author that blood should not have been effused at the moment of the accident. In a young man, in the highest degree of animation, coagulation of the blood would so immediately follow an injury in a part, the actions of which are so constant, as to arrest the flow of blood beyond the small quantity sufficient to tinge the serum found in the pericardium. We know as yet too little of the resources of the animal economy, to ascertain what might have been the issue had the ball passed quite through.

II.—*Instance of a Disease producing Hydrothorax and Anasarcous Dropsy, with constant Expectoration, copiously, of purulent Sputum, for longer than a Year, without Abscess or Ulceration of the Lungs.* Extracted from a Clinical Lecture, by George Pearson, M.D. F.R.S. Senior Physician to St. George's Hospital.

This case is worth recording, and is well related. That purulent sputum, or pus, should be secreted without any breach of the solids, and particularly on mucous membranes, is a fact well known. The origin of this disease is not more obscure than many, we might say, most others, among labouring men. They continue, from ignorance and supposed necessity, their employments, when they should have immediate relief. Hence, the consequence of an acute disease, which might have been subdued, is often a chronic sequela, calamitous and fatal. Such appears to have been the case with this man. Anasarca is a very frequent attendant on pulmonary complaints of every description, and is generally conjectured to arise from an imperfect oxygenation of the blood. There were, however, in this case, many circumstances which might have led to suspicion of inflammation, and this suspicion was not contradicted by the effect of the various remedies applied. We wish so accurate a chemist had analysed the urine during life, and the effusions found after death.

III.—*Observations on Blood-letting from the Hæmorrhoidal Veins in certain Cases.* By William Brown, M.D. Fellow of the Royal College of Surgeons, Edinburgh.

The great object of this paper, which is well written and extremely well directed, is to lead the practitioner to local bleeding, particularly in the hæmorrhoidal vessels and their neighbourhood. The following passage will explain the author's meaning :—

"It appears (says he) somewhat odd, that, while topical congestion of blood is admitted to exist in a variety of cases, the way in which it is thought this accumulation can be best removed is by taking blood from the arm. One would think that the most effectual way would be to empty the vessels themselves immediately; and, farther, that we should follow the course we observe the system pursue to relieve itself. The fact that the head is eased by a bleeding from the nose, and the lower belly by the bleeding-piles, is indisputable: nay, that a small quantity of blood from these parts will give more relief than a larger quantity taken elsewhere."

Now, local bleeding is at present more practised than at any former period. Cuppers are increasing in number, and each increasing in wealth. But we must be careful how we confound local inflammation with congestion, or expect the same advantage from abstracting a small quantity of blood under inflammation, as the constitution feels by the spontaneous bleeding from the nose, or hæmorrhoidal arteries. In the latter case, the whole system is relieved by the very local congestion. If no blood escapes, by degrees the congestion will cease ; and, if in the parts above-mentioned, neither they nor the system may suffer any material injury. A small quantity of blood escaping seems to give great relief, but this appearance is often fallacious. If it escapes at that period when the local plethora is likely to cease, it will have the credit of producing all the relief the patient feels. But that this is not the case, we have a sufficient proof in two very familiar instances. Immediately before menstruation, all the parts in the neighbourhood of the uterus become more turgid: the mammæ at the same time. In this condition they continue for several days after menstruation has ceased, and then gradually subside, whether the menstruation has been copious or not. In the same manner, persons who suffer an almost periodical discharge of blood by the rectum feel always a previous fullness in the whole abdomen, which terminates in a similar manner. Still, however, we should think it wrong to omit the description of a practice which we doubt not may, on many occasions, be attended with advantage, and the mode of conducting which must be approved.

"It has been alleged, and perhaps not without reason, that we are disposed to hold foreigners in too little estimation, and therefore are unwilling to be taught by them. Indeed, the exertions which Great Britain has made in science, arts, and war, give her some title to assume a tone of superiority. Yet it is true that, in some things, she is behind countries which she considers as almost barbarous. She might learn more things from them in the arts and conveniences of life. Among others, I think she might be benefitted, without degradation, by attending to the practice of the old continent in the case which I have endeavoured to state.

"The advantage of taking blood from the hæmorrhoidal vessels is so well established in many parts of Europe, that the practice is, in a great measure, taken out of the hands of the medical profession. Hence, in some places, there is reason to think it is often employed without necessity, and is productive of inconvenience, by establishing a habit not easily to be overcome. The operation necessary is committed to male and female practitioners, who execute it with surprising dexterity, by the application of leeches to the verge of the anus. The patient is subjected to no indelicate exposure, and to very little trouble. If strength permits, he is seated on a perforated chair, which only uncovers the anus itself: the operator, stooping or kneeling, by means of a taper, sees the part to which the leech is to be applied; and, provided with a small round wide-bottomed bottle with a long neck, just large enough to contain one leech, he allows the animal to crawl out and fix itself on the part intended. The operator, having applied one leech, withdraws the bottle, and proceeds to fix one after another till the desired number have been applied; a basin is placed under the chair, into which the blood flows. The discharge of blood is promoted by the use of a warm sponge, which gives also the patient the advantage of a warm fomentation. If the sick is unable to get out of bed, the exposure and fatigue of this remedy are not greater than what accompanies the administration of a glyster.

"I have stated that this practice is often precautionary, and occasionally abused; but its employment is more frequent in cases of acute and dangerous disease. It is a most powerful auxiliary to general blood-letting, and at the same time a means of rendering a great evacuation of the vital fluid much less necessary. In cases of abdominal inflammation, such as hepatitis, enteritis, puerperal fever, in suppressed menses, lochia, &c. this cure is put in practice with the greatest success. Instead of applying leeches to the parietes of the abdomen, which is sometimes the case with us, they place them on the hæmorrhoidal veins, and uniformly find they more effectually, because more immediately, empty the vessels belonging to the abdominal viscera."

IV.—*Case of Lithotomy*. By T. SMITH, Surgeon, Kingussie.

The patient was 77 years old, had an inguinal hernia, and, after the operation, suppuration and a swelled testicle. The operation was repeated, a stone having been afterwards discovered by a search made whilst the hernia was reduced. The slightly-adhering wound was re-opened, and two stones extracted, whilst the hernia was kept in the abdomen. It appeared that the protruded hernia so affected the form of the bladder, as to place the stones out of the sphere of contact of the sound. The operations were performed with the scalpel.

V.—*A concise Account of the Typhus Fever, at present prevalent in Ireland, as it presented itself to the Author in one of the Towns in the North of that Country.* Being the Substance of a Paper read before the Royal Physical Society of Edinburgh, in November 1817; by WM. L. KIDD, M.R.C.S. London, President of the Royal Phys. Society, Edinburgh, and Surgeon, Royal Navy.

The rise and progress of every disease is highly important, and every document serves to render it better understood. It is now very universally admitted, that unfavourable seasons, bad and insufficient nutriment, have been the cause of the fever which has induced so much distress in our sister kingdom. The history given by Mr. Kidd coincides with what has generally been admitted,—that it every where began among the poor, and spread among them; that, when the rich were affected, it rarely spread, but oftener proved fatal; that, in all, evacuations of some kind were found useful, but with much greater necessity and freedom in the wealthy. We much regret the author's adherence to the term Typhus, the definition and treatment of which by Cullen is altogether inconsistent with his (the author's) account of the most successful mode of treatment, whether medicinal or dietectic. The following passages will best explain our meaning.

"After what I have said in the former part of this paper, it will, no doubt, appear strange that it was in the cases of the poorer classes of society that I was enabled to give the most favourable prognosis; but, however strange, it was not less true. Poor and squalid, wretched, and unprovided with every comfort, as they were, their recovery was by far more probable than that of those who knew no want of either comfort or attendance. Often have I seen two, and not unfrequently three or four, of those miserable creatures huddled together, without regard to age or sex, in the same bed, covered with every thing that appeared noxious and disgusting, and in want of the only covering that would have been considered as likely to promote their recovery,—by means of a very little medicine, and a very little attention, recover their perfect health, and in a short time be able to pursue their usual occupations: while those who possessed every thing that appeared necessary to their state, or likely to facilitate their recovery, either speedily fell victims to the disease, or suffered all the miseries of a protracted recovery, constantly threatened by, and often sustaining repeated, relapses.

"This difference in the violence of the disease, or what perhaps I should be more justified in calling a difference in the termination of it, I was unable to account for in any other manner than by supposing, that, although the low and unwholesome diet to which the poor had been for several months restricted, was evidently one of the principal causes of their illness; yet that "out of that thorn *poverty* they had plucked the flower *safety*," by being thereby, in a degree, prepared for the disease, and reduced to a certain state even before it had made its attack, which enabled them the more easily to elude its grasp, and to which state it required several days' illness to reduce the frame of the more affluent, the better fed, and consequently the more robust; so that, to use a common figure, the disease passed through the people as a hurricane through the forest, which merely bends for a time the pliant willow, but uproots the unyielding oak. If, however, we admit, with some authors, that there are two kinds of motion in the constitution of a fever,—one noxious, and possessing a tendency to hurt and destroy the body, and at the same time producing in it certain motions which deviate from the natural state; the other of a salutary tendency, produced by the operation of the *vis medicatrix naturæ*, tending to obviate the effects of the noxious power, to correct and remove them;* which latter is called the re-action of the system, if indeed the latter can be called a part of the disease: it will be easy to account for the favourable termination of the fever in those who, by bad and scanty diet, were reduced to a state too low to be capable of a powerful re-action; and why the termination was more generally unfavourable in those whose constitutions and frames were preserved in full vigour, by means of a free and generous diet, up to the very hour in which the fever began to manifest itself."

"On being called to a patient, and on ascertaining that the disease really was typhus, the treatment varied, of course, according to the symptoms present, and according to the space of time which had elapsed from the commencement of the fever. When called in at the very onset of it, I have frequently succeeded in putting a stop to it altogether; or, if not, to arrest its progress considerably, by the administration of a combination of the sulphate of magnesia and of the tartrate of antimony, dissolved

* Vide Cullen's First Lines, lib. i. c. ii. § 46, and c. iii. § 59.

in a large quantity of water; a small dose given every hour, and repeated for twelve or fourteen hours, let the effects produced be what they might, unless they should prove unusually violent. In this medicine, the violent effects which are often observed to follow the exhibition of the tartrate of antimony are completely prevented by the large proportion of water in which it was dissolved, by the smallness of the dose, and still more by the disposition which the sulphate of magnesia gave it to affect the bowels. In these cases, then, the effects generally were—moderately emetic, fully purgative, and mildly diaphoretic; and in one or two, or sometimes in all of these ways, it appeared to produce the most beneficial effects. During the operation of this medicine, I had the patient's skin well cleansed, and generally relaxed by a free washing with warm water and soap, which was repeated the same day, or on the following morning. Mild tepid drinks were taken while the stomach and bowels continued to discharge their contents. After these had been thus freely emptied, I have frequently had no occasion for any further medicine; but, in despite of the use of these means, the disease would occasionally continue its course. In that case, the skin becoming hot and dry, the head, feet, and legs particularly so, I had recourse to the application of cold water to the surface, —not, indeed, to the extent I could have wished, but as far as I could prevail on the friends of my patients to admit of its use; which never went further than washing with cold water. In some cases I succeeded in having this generally made use of; in most, however, it was only topically applied: in some, the water was perfectly cold; in others, I was obliged to submit to an admixture of warm. In some, where the cold ablution would not be admitted, I compounded for the application of cloths wet with cold water being applied to the surface, and frequently renewed. This practice I found to be of the utmost service, when the cloths were applied in this way to the head, which I generally caused to be deprived of the hair. Vinegar, or vinegar and water, were usually made use of as topical applications to the head; and cloths wet with these were incessantly applied, and renewed frequently, so long as any unusual heat or tendency to delirium manifested themselves. If, however, the affection of the head did not soon appear likely to give way to this mode of treatment, I had recourse to my lancet, and opened the temporal artery; which generally proved successful. In one case only did I apply a blister to the head; and it was not done until, by two days' solicitation, I had been teazed into compliance by my patient, who was a surgeon: and in that case I regretted having done so, as it prevented the continuance of the cold application to the head, which I am confident would have produced more benefit. In fact, there were very few of the patients under my care whose heads became very seriously affected; but I knew of some in which all the symptoms of phrenitis occurred. This freedom from disturbance of the brain to any alarming degree in my patients, I mainly attributed to the cooling plan which I pursued, for, in some cases, among the very lowest classes of the poor, where, on visiting and finding the patient quite delirious, I gave directions for opening all windows, doors, &c. in order that a free current of air might be established, I have frequently found, on repeating my visit in a few hours afterwards, that the delirium was either totally gone, or much abated. To many of my patients the application of cold water became very agreeable, and some of them became urgent for a frequent repetition. Its most usual effect was to relax the pores of the skin, and to change the harsh dryness and pungent heat of the whole surface into a soft and gentle diaphoresis, to remove or diminish the distressing watchfulness, and to induce a quiet and refreshing sleep."

These and the other remarks are only a confirmation of Dr. Jackson's practice; but most of all, of one caution, which, we believe, originated with that physician, and of which his brethren took so ungenerous an advantage as to accuse him of protracting the recovery, and even increasing the number of relapses in our brave defenders, from mere motives of parsimony. The following paragraph will explain our meaning.

"The convalescent stage (says Mr. Kidd) I found a very difficult one to manage, and relapses were very common. This appeared particularly in those whose health and appetite for food returned more suddenly, than among those whose recovery appeared more lingering; I, therefore, became particular in restraining the appetite of the former, as well in the quantity as in the quality of their food, and in paying a scrupulous attention to the state of their bowels."

We sincerely join with the author in his regret that the prejudices of the country would not permit any examination *post mortem*.

VI.—*Case of Typhus, attended with Trismus and Amaurosis.* By John Astbury Barlaston.

VII.—*Observations on certain Dropsical Affections which are successfully treated by Blood-letting.* By J. Abercrombie, M.D. Fellow of the Royal College of Surgeons, Edinb.

This is an ingenious and well-written paper; but we should hardly have thought it necessary to dwell on a fact now so well admitted, that we have sometimes doubted whether it might not be carried too far. Dr. Blackall has (as the author of the paper remarks) thrown great light on this subject; so much, that we should conceive a reference to Diocassus, Hildanus, and several others, the less necessary. But, considering the well-deserved reputation of Dr. Parry, we are doubtful whether the practice may not become too general. Dr. A.'s paper concludes with a very necessary caution concerning the uncertainty of the pulse in these diseases, illustrated with a strong case.

"I have stated, (says he,) that, in the treatment of affections of this nature, the strength of the pulse is a very uncertain guide; for, when the transmission of blood through the lungs is much impeded, we often find that the pulse is small and even irregular, and that it improves in strength, and becomes regular, after copious blood-letting. The following example of this has occurred to me since these observations were written.

"A stout young man, a mason, aged about 28, was affected with slight anasarca, cough, and uneasiness in his breast. The latter was most troublesome in the night, when it amounted to dyspnœa, with a sense of constriction across the thorax, and prevented him from lying upon either side. His pulse was from 70 to 80, weak, and very irregular. He had been ill about a week, during which his nights had been passed with much uneasiness, but through the day he had been able to attend to his business.

"He was bled to ℥xxiv."

VIII.—*Some Observations on the Treatment of Phlegmon.* By RICHARD LANPHIER DRAPER, Surgeon, &c. &c. in Ross, county Wexford.

IX.—*An Account of two Still-born Children, restored to Life a long time after Birth.* By HENRY TERRY, Member of the Royal College of Surgeons in London, &c.

These cases are well worth recording, but exhibit nothing more than what should be the duty of every practitioner, and what he ought to attempt under similar circumstances.

X.—*Report of Diseases on board the Bellerophon.* By ARCHIBALD ROBERTSON, Surgeon.

XI.—*Observations on Medical Legislation.*

XII.—*On a particular Fracture of the inner Condyle of the Humerus.* By BENJAMIN GRANGER, Surgeon, Burton-upon-Trent.

This is an useful practical paper; probably, we may hereafter transcribe it into our Collectanea.

XIII.—*Observations on the Cure of Syphilis without Mercury, and on a peculiar Affection of the Alimentary Canal, sometimes mistaken for Syphilis.* Communicated in a Letter to Dr. Duncan, jun. By JOHN HENNEN, Deputy Inspector of Hospitals.

After the very long paper contained in our last Number but one, we should have thought it unnecessary to return to the subject, were it not again to remind our readers how carefully they should examine an author before they quote him. With the generality of books this may be less important; but, in a writer of such authority as Mr. Hunter, and who is said to be comprehended with so much difficulty, surely something more than a superficial glance seems necessary.

To do justice to Mr. Hennen, we shall transcribe the introductory part of his paper, as it introduces the name which, in spite of all the multiplied objections to his doctrines, is, most unaccountably, the only standard to which his numerous opponents refer us.

"In the present state of the question of the treatment of syphilis without mercury, it would be quite superfluous in me to make many remarks. The results of several trials have already been laid before the public, as they have occurred in the military hospitals of this city, by Dr. Thomson; and in those of London, by Messrs. Guthrie and Ross.

"I have felt great pleasure in showing you such of the cases as were still under treatment; and I know that I fulfil the wishes of my respected chief, Sir James M'Gregor, the director-general of the medical department of the army, when I solicit the inspection and opinions of medical practitioners, who are as well able to form a judgment as yourself. And I beg to assure you, that the same spirit of candour which has induced me to show you the evidence upon one side of the question, will point out to me, as a duty, to communicate to you every thing which may hereafter appear upon the other. But as some misconceptions may arise in the minds of those who hear of, without being able to see, the practice to which a fair trial is now giving, I think it necessary for me to trouble you with a few observations.

"It has been supposed by some persons, that the cures effected apparently without mercury, have been actually performed by means of the different preparations of that mineral, and by caustic surreptitiously employed. But the slightest acquaintance with the discipline of military hospitals, as at present conducted, would point out the impossibility of such a practice.

"It has been urged, that the primary sores which have disappeared under the antiphlogistic regimen, cleanliness of the parts, and mild local applications, have been mistaken in their nature. On this I beg to remark, that, in the half-yearly returns of diseases sent in from the different military hospitals to the head of the department, there are no less than five separate columns appropriated to the classification of venereal complaints, their consequences and concomitants, exclusive of gonorrhœa. Among these, there is a special head for the "ulcera penis non syphilitica,"—a class under which, as is well known, a great variety of morbid affections of the genitals range themselves; but which, having been very frequently confounded with true syphilis, and at once submitted to a mercurial course, have not been allowed to evolve themselves, but have had their nature totally changed or modified by such treatment.

"That these sores, (at least all of them that hitherto have appeared in the military hospitals here,) and also that the species which Mr. John Hunter has designated as the true syphilitic sore, heal without the employment of any other means than rest, abstinence, cleanliness, &c. is perfectly demonstrable, and is daily to be seen in the wards at the Castle, and at Queensberry House, appropriated to such cases."

Now, it unfortunately happens that Mr. Hunter has described no true *syphilitic* sore on the penis, and seems to doubt whether they ever exist on that part. The term syphilis he confines to *secondary* symptoms. To ascertain primary venereal ulcers, or chancres, it will be necessary to study his account with more diligence than is usually done. When this is accomplished, the reader will cease to wonder if such sores cannot be ascertained to be chancres, till they have had all the means of "rest, abstinence, and cleanliness;" after all which, only their true character is "demonstrable."—[*See our Number for March last.*]

Connected with the last article, we consider the following paragraph in the Medical Intelligence of this Number.

"*Solecism.*"—"How many years further must the correct taste of physicians be offended by the use of the terms *morbid anatomy*, *morbid poisons*, &c.? What is the meaning of these terms? No doubt, they must signify what is meant by the words *diseased anatomy*; for, surely, *morbid* and *diseased* have the same import. The words *morbid poisons*, in course, have the same signification as the terms *diseased poisons*. In these instances, the notions intended to be expressed, would be justly communicated by saying,—*anatomy of diseased parts*; *anatomy of diseased lungs*, *eye*, &c.; *poisons which occasion diseases, or morbific poisons*. These improprieties did not escape the acuteness of a good philologist, the late Dr. Beddoes, and it was reasonable to have expected that the disuse of them would immediately have taken place on their exposure."

As we have always professed a great attention to language, so we shall always be thankful for any instruction and correction on this very important part of philosophy, because the slightest inaccuracy may be followed by errors extending we know not how far. First, then, what is meant by a *solecism?*—We are told, by grammarians, that the term originated in the very impure dialect of the Soloeci, who inhabited a city in Cilicia, and spoke a mixture of Attic and their own dialect: that hence every expression which came under this description,—that is, that was not entirely barbarous,—acquired the epithet of *solecism*. If this is the case, we conceive the accusation comes with an ill grace from a provincial physician; or even from the inhabitants of a metropolis in which the English language is imperfectly spoken. We admit that the word *morbid* is Latin: the Scottish editor has, however, thought proper to translate it into English, and we scruple not to say that the translation is incorrect.

As the acuteness of that "good philologist, the late Dr. Beddoes," is here referred to, we shall first refer our readers to the passage in which his remark is to be found, and also to the answer. Dr. B.'s complaint of the danger lest the "phrases *morbid poison* and *morbid anatomy* should become inveterate," will be found in our 20th Volume, No. 117, p. 427 and 428. The answer is in the same volume, No. 118, p. 535 and 536. Dr. Beddoes was too cautious to substitute any other term for *morbid poison*; and, probably, for this reason, he only hints at, without transcribing, Mr. Hunter's "*ill-contrived phrase.*" To Dr. Baillie he shows no mercy in any way, and avails himself of the French translation—*Pathological* Anatomy. To this term, we make no objection, though, in strict hypercriticism, it might be shown to be as deficient as the former: but, *pathological poisons* is too bad for either Dr. Beddoes, or the Edinburgh critic, and the latter has thought proper to adopt a term to which we recently stated our decided objections. We are particularly obliged to him for his candour in translating the phrase—*Poisons which occasion diseases*, or *morbific poisons.* Now the next thing is, to explain what those *poisons* are *which do* NOT *occasion disease.*—Here we confess ourselves entirely at a loss.

That the English language is deficient in adjectives, is a fact universally felt, though not so universally complained of. As a celebrated etymologist remarks, our nouns are so ill calculated to form into adjectives, that we frequently use the noun without any alteration in its termination, either as a substantive or adjective—this we shall presently see in the word *animal.* At other times, where we have a different termination, we consider it a matter of indifference;—thus we say, *a gold watch*, or *golden watch; a wood-cut*, or *wooden cut.* When our ancestors wished to form an adjective, whether to one of their own nouns, or to one derived from the learned languages, they frequently chose to go back to a language better suited to such change of termination. Thus, for the commonest words, *man*, *woman*, and *boy*, they felt the convenience of *human*, *femenine*, and *puerile;* and, in *giant*, they found it absolutely necessary to go back to the original for *gigantic.* We suspect that even the title to the respectable work from our sister metropolis, which we are now noticing, has no authority higher than Mr. Abernethy.—Every former writer thought it necessary to make *chirurgical* the adjective of *surgeon*.—By degrees, the term *surgery* acquired some authority, and we are far from objecting to *surgical*—conceiving, that where the sense is not obscured, there can be no impropriety in rendering our language as uniform as possible.

But to return to *morbid poison*.—That some distinction should be made between such animal poisons as are secreted in venomous animals in *health*, and such as are only the effect of *disease*, can hardly be questioned ; and, it is not less certain, that *morbific* is an inadequate term, inasmuch, as it comprehends not only every animal poison, but also every vegetable and mineral poison.—Besides, as we before observed, the term is superfluous, inasmuch as it is comprehended in the word *poison*.

It may then be asked, would not the term *animal* poison be sufficient of itself? We answer, no—because the want of some distinction between these two kinds of animal poisons has led us into error. Celsus, in his arrangements, places them all under one chapter :* he has been followed by most authors, and the consequence has been, that similar remedies have been applied to each, as may be seen in Celsus and in the once-popular Treatise of Dr. Mead.

Mr. Hunter, whose talents seem better appreciated any where than in his native country, first saw the necessity of this distinction ; and for this he might have claimed a share of merit, even had his terms been somewhat objectionable. But we scruple not to say, that we can find no objection to the term, and that *morbid* and *diseased* have not the same meaning. Dr. Beddoes was so well aware of this, that he did not choose to translate *morbid* into Latin by the word *morbidus*, knowing that the Latin word had a more general meaning than *diseased*. He was, therefore, uncandid enough to translate the English word *diseased* into *ægrotans* and *νοσȣσα* ; by which he rendered himself absurd, to say the least. Though we have no such verb as *to disease*, yet *diseased* is evidently, by its termination, and by its constant import, the participle of the verb which should be formed by *dis* and *to ease*. *Morbid* has no direct reference to any verb, nor has it a participial termination: it means, therefore, only, as our lexicographers explain such words—*of or belonging to disease*. In this manner *Morbidus*

* Lib. v. cap. 27. De vulneribus quæ per morsus inferuntur, eorumque curationibus.

is used by the best Latin writers. See Pliny and Lucretius: the latter uses the terms—*Vis Morbida; Morbidus aër.* How would these phrases read, if *morbid* must be always interpreted *diseased*, or *ægrotans*, or *νοσεσα*?

Thus, as *morbid* may comprehend generally every thing *of or belonging to disease*, we must consider it the most appropriate term, till we are favoured with a better, for those animal poisons which originate in diseased action.

---

*Medico-Chirurgical Transactions, published by the Medical and Chirurgical Society of London.* Vol. VIII. Part II.—Longman and Co. 1817.

(*Continued from p.* 327.)

*Three Cases of Calculi, removed from the Urethra without the Use of cutting Instruments.* By ASTLEY COOPER, Esq. F.R.S. Surgeon to Guy's Hospital.

It is not a little remarkable that, on the revival of this practice, the case related nearly half a century past (by Mr. Warner, we believe, but have not at present leisure to turn over the Transactions of the various Societies,) should be so entirely overlooked. The consequence of successfully dilating the urethra in that case was, however, that it never sufficiently contracted afterwards to enable the patient to retain her urine. In the case first related in the paper before us, this inconvenience existed before the dilation, and continued afterwards. The condition of the second, after extraction of the calculus, is not mentioned. In the third, the urethra contracted, and the patient, a girl only eleven years old, was completely cured.

Among the remarks of the author of the paper are the following.

"A great advantage will result from this mode of operation, if it should be found that, in the majority of cases, the urine is retained after the extraction of the stone; as the great objection to the use of the gorget or knife, in the operation in the female, is the loss of power of retention which follows it, leaving the patient offensive to herself and friends, and the subject of continued excoriation. It is true Mr. Hey has suggested the introduction of a sponge into the vagina, in the hope that, by the constant application of the surfaces of the wound to each other, they might be made to unite; and, when cutting instruments are employed, such a trial will be proper.

"Another advantage will be derived from this plan, viz.—that it may be employed as soon as a small stone is discovered in the bladder, when it can be extracted with great ease, and at a time that a more dangerous, important, and painful, operation would be hardly proposed."

To the last of these remarks, we can have no objection; but we must receive several more reports before we are satisfied that, in adults, the urethra will with certainty sufficiently contract after any extraordinary dilation, continued for several days. It is true, as Mr. Cooper remarks, that the same inconvenience often happens after the common operation, attended with considerable pain, and other ill consequences. We know not with what success Mr. Hey's plan may have been attended; but this we know, that the continental surgeons usually introduce a flexible catheter during the high state of inflammatory adhesion soon after the operation; and we have been assured by Professor Scarpa, that, by these means, the wound, for the most part, heals, without being followed with such dreary consequences.

*Some Cases of Diseases of the Heart, with an Inquiry into their Nature and Causes.* By J. H. JAMES, Esq. Surgeon to the Exeter Hospital. Communicated by Mr. Abernethy.

Without mentioning names, which we conceive unnecessary, we may be allowed to remark, that many among the most distinguished teachers of the present day have an organ which they ride, if not unmercifully, at least almost unremittingly. Yet all these hobbies are terrible jades. With some the liver is constantly foundered; with others, the digestive organs; with others, the head. At length, the heart is now brought in to share the burthen and the blame, not, indeed, without some sympathy; for we shall find that she often suffers from the failings of other parts, whose duty it is implicitly to obey her.

It is difficult to do justice to a paper occupying nearly sixty pages: we shall, however, transcribe the introductory part, slightly touch on some of the cases, and offer a few remarks on these, and on the author's general conclusions.

"In the writings of the older authors, there are numerous cases recorded of examinations of persons who have died with diseases of the heart; many of them highly valuable, but few complete, inasmuch as the views entertained of this, as well as other subjects, were different in many respects from those of their successors: perhaps I may be warranted in saying, because they were less enlightened; and also because they have, in many instances, omitted to give us a sufficiently detailed account of the appearance of other organs besides those principally concerned. In the works of several modern authors, there are cases related of much more value, from the greater care and fidelity of the examinations, and the more instructive views which they present. An addition to our collection of facts in this department is still, however, very desirable; and I am, therefore, induced to lay the following cases before the Society. They are selected from among those which I had an opportunity of observing, particularly during the latter part of the period that I passed in attending St. Bartholomew's Hospital, when I had very extensive opportunities of seeing the morbid appearances after death, as well as observing the symptoms before it; and my attention to this class of diseases was particularly excited from the frequency with which these cases occurred.

"From the facts which I observed, I was led to form some opinions respecting them, which are in many points, I believe, peculiar; and, although in the interval which has since elapsed, I have not been fortunate enough to have it in my power fully to confirm them, yet I have not seen sufficient cause to reject them as erroneous.

"Whenever mechanical obstruction has been found to exist at either of the orifices leading from the cavities of the heart, some of these cavities have been found to be enlarged in size, and very generally strengthened in structure: one or more may have undergone this alteration, but, most commonly, we find that cavity altered in particular, before which the obstructing cause is placed. Every one, therefore, has agreed to consider this as one of the chief causes of aneurism of the heart. But, if contraction of the aorta at a distance from the heart exists, enlargement of that viscus, it is supposed, may equally take place; or this, indeed, may happen if obstruction be situated in any other part of the vascular system. Of such cases there are some on record;* but a very few where there is positive evidence of the fact; and I am therefore particularly induced, in the first place, to offer the three following. In the first, there was evidence of obstruction in the course of the vascular system, and the heart was enlarged, but there was also obstruction at the aortic orifice: nevertheless, I am inclined to believe that this latter was consecutive. In the second, there was evidence of a contracted state of the aorta, with enlargement of the heart. In the third, there appears to have been obliteration of the vena cava, likewise causing enlargement of the heart. Independently of these points, there are some interesting circumstances belonging to these cases."

We are ready to admit, that a true pathologist will find something interesting in every case of morbid dissection. As, however, Mr. James has given an epitome of these cases,

---

* "Morgagni, Book ii. Letter xviii. art. 2.
"Corvisart, Obs. xxiv. p. 117.
"These are the only cases of this kind I find in two works very copious in cases of diseased heart.

we shall only notice the last, with his remarks. The body, we are informed, was opened soon after death.

"The pericardium was much distended with serum. The heart gorged with blood. All the cavities, the coronary veins, and the great vessels, full.

"The right ventricle was thin: the left very strong in its structure.

"The abdominal aorta was empty; its calibre was nearly the same as that of the vena cava at the same part; but the artery was capable of being stretched to a considerably greater extent, while the vein did not admit of being stretched at all: so that it is pretty clear that, in this instance, the artery was capable of transmitting much more blood than the vein.

"Through a considerable space the brain was crammed full with a firm coagulum, which occupied the whole diameter of the vessel, and left no interspace whatever between it and the sides, which were at the same time thicker than usual. It was not a coagulum equally tinged with red particles throughout, or altogether without them, as in what have been mistaken for polypi of the heart; but more of these particles were contained towards the sides all round, while the centre was nearly white. At the time this coagulum was observed so firm in the vena cava, the blood in the other veins was fluid.

"Several parts of the internal coats of the artery had a deposition of lymph, which could be scraped off. I think the internal coat was thickened—the middle one was so decidedly.

"In the head, great effusion was found between the tunica arachnoidea and pia mater.

"I think it will not be considered a very improbable conjecture to suppose that, whatever the nature of the disease might be, there was decided obstruction to the return of blood to the heart. And it is also highly probable that the principal seat of this obstruction was in the vena cava. It cannot be proved that the coagulum, which filled that vessel after death, had existed during life; but it is highly probable, from the appearances, that it did. The coagulum found was by no means like that which usually takes place spontaneously in the blood, which is never so firm and entirely solid as this. Besides, why should the blood have coagulated in the vena cava only? From the lymph effused on the surface of the arteries, as well as from the peculiar state of the vena cava, it is not improbable that there was diseased action of the vessels generally."

On the above we would only remark, that this difference in the extensibility of the two great vessels is nothing remarkable. It is universally known by those who attend closely to the formation of parts, that the aorta may be made more capacious than the cava. Such a provision for the aorta, which has to receive, at an angle, the full impetus of the left ventricle of the heart, is absolutely necessary. That it is rarely, if ever, completely extended, must be known to every one accustomed to experiments on living animals. Should, however, a larger portion of blood be thrown out by the aorta than the capacity of the cava will return, it is well known that the venous system is much more comprehensive than the arterial; besides the provision made in the sinuses of the brain, and in the great dilatability of the external jugular vein. In the subject just referred to, the probability is, that, *in articulo mortis*, when the muscles all became stiff, the arteries contracted so as to throw all the blood into the veins and the heart. If this was the case, there is nothing very remarkable in the appearances described. The coagula showing a separation of lymph from the red particles is not uncommon. (See Morgagni and others.) We are, therefore, by no means satisfied with the following conclusion of the author, even if we perfectly understand him.

"In these cases—certainly in two of them—there was evidence of mechanical obstruction, situated in the course of the circulation; and I presume that the enlargement of the heart may be attributed to this cause. In that which follows, it also appears probable that obstruction existed in the course of the circulation in the great vessels; however, at any rate, not *mechanical*, but *functional*."

In a subsequent case, Mr. James informs us—

"On the following day, May 14th, his body was examined, fifteen hours after death, and it was then still warm. The face was suffused with blood; the jaws strongly clenched; and there was a universal rigidity of the muscles."

The heart distended the pericardium; it

"was very large, and its cavities were all gorged with blood; so were the venæ cavæ, pulmonary artery, veins, and aorta. The cavities of the right side were merely enlarged and filled with blood, but the left ventricle was not only enlarged in capacity, but its muscular structure was also greatly increased. The aorta was enlarged generally, and there was a great deal of ossification about the root of it. The valves were also ossified in some degree, but not so as to prevent them from performing their functions properly. In the thoracic aorta were also some specks of ossification, and in the abdominal it existed to a very great degree indeed.

"In this case there was evidence during life, as well as after death, of augmented force of the action of the heart, and under distention of the arterial system. Analogy would lead us to suppose that it proceeded from obstruction; but there was no organic alteration of structure about the heart itself likely to produce it, and it is therefore fair to suppose that the obstruction was situated in the minute vessels, of a very different nature, perhaps, from that which existed in the case last related.

"Was the ossification and disease of the aorta an idiopathic disease of the vessels? or was it produced by the increased actions of the vascular system? This disease is so common a concomitant of active aneurism, that it is difficult to avoid supposing it to be either a cause or an effect; but if the former,—that is, if it be idiopathic,—why does it not as often occur in the pulmonary artery? If it be considered as a consequence of the disease of the heart, this difficulty is easily explained, because that disease is found on the left side more frequently than on the right, in the same proportion, probably, as disease of the artery which leads from it. If we should be able to explain hereafter how it happens that disease of the heart so much more frequently occurs on the left side, then we may at the same time account for the ossification of the *systematic artery* being so much more general.

"Where there is an enlargement of the ventricle, and augmented strength of pulsations during life, increased capacity and strength of the arteries is found to exist after death, especially if the case has been of long standing; and also, very commonly, ossification and steatomatous thickening. That the artery should be enlarged in the same proportion as the ventricle, seems natural enough; and also that its parietes should be thicker, since it has to sustain the impetus of a larger column of blood, impelled with preternatural force; but, if there be a difficulty opposed to the arteries in emptying themselves of their contents, which I imagine to be the case, it will follow that the vessels which are now immediately in the neighbourhood of the principal trunk, will have, more especially, the blood forced into them with undue violence; and this will be very likely to occasion unhealthy deposition in the parts so supplied. The vasa vasorum will be particularly exposed to this cause of derangement.

"The coronary arteries in this case, as in most of a similar nature, were also greatly enlarged. If we suppose obstruction to the passage of the blood at the extremity of the arterial system, the resistance would, of course, throw back the blood to the heart more particularly, as being the other end of the column. But, at the orifice of the aorta, valves are interposed to prevent their reflux. In addition, then, to the first and natural impulse caused by the action of the heart, there will be the re-action occasioned by the obstruction, and the effects of this will be more felt near that viscus. It is not, therefore, to be wondered at, that the coronary arteries are found to participate, in an eminent degree, in the alteration produced by this state.

"Corvissart has considered this augmented influx of the blood into the heart as one of the causes of active aneurism. That this opinion is erroneous, I think I shall hereafter be able to prove; but this is a question foreign to my present purpose."

Several other remarks follow; but we cannot dismiss this passage without some observations. The body was opened fifteen hours after death, was *still* warm, and universally rigid. Every one accustomed to watch the progress of death must know that the expression *still warm* is liable to be misunderstood, because the warmth then perceived was not the effect of heat remaining from the period when respiration first ceased, but a process which began probably some hours after, at the time when the stiffening commenced. We may, perhaps, have more to say hereafter concerning the *systematic* artery, M. Corvissart, and active aneurism.

The author only collected his accounts of the following case after death, and, as far as we can perceive, only from the nurse and the apothecary's book: it is, however, well worth comparing it with the symptoms.

"Case VI.—May 6, 1812, I assisted at the examination of the body of William Holford, who died in St. Bartholomew's Hospital: the following were the appearances observed.

"On opening the thorax, the lungs and pleura were found perfectly healthy, and there was no effused fluid. The bronchial glands were enlarged and hardened. The mucous membrane lining the bronchiæ rather red. There was a great deal of fat in the mediastinum and about the heart. The heart felt very large through the pericardium: on opening that membrane, it was found covered with lymph, which was more particularly heaped up about the roots of the great vessels; there were no adhesions. The cavities of both the auricles were larger than common; of the ventricles, about the usual size. The muscular structure of the right ventricle was of its ordinary strength: of the left, was most enormously increased in size and strength. The aorta unusually large and strong; the coronary arteries much larger than common; the aortic valves quite healthy.

"The abdomen was not distended, but the caput coli was found to occupy a large portion of it. The œsophagus at its lower part, for six inches, was inflamed, and lymph thrown out. The stomach also had some appearances of inflammation: it was filled with matter apparently fæcal, much resembling what was found lower in the alimentary canal, and properly tinged with bile.

"The colon was distended, in two or three places, into enormously large pouches filled with air, and in the intermediate space was contracted to a very great degree; contracted upon fæcal matter, hard almost as bullets, which appeared to have remained in these pouches a considerable time,—for, where the scybala were lodged, the coats of the intestine had become exceedingly thin; as if the contraction of the muscular coat had, by pressing the mucus against them, caused it to be thinned by absorption. There were large masses of these hardened lumps even in the rectum; but there was in no part any of that diseased secretion, which often constitutes diarrhœa where there are scybala.

"The liver seemed perfectly healthy. The peritonæum was not inflamed; there was a good deal of fat deposited in every part of the abdomen, and there were a great many adhesions. The bladder contained a considerable quantity of healthy urine. The kidnies were very small, and much diseased: the renal arteries unusually large.

"This man had lost much flesh, but his fat did not appear to be wasted. He had sores upon his legs.

"He was opened shortly after death: the corpse had a strong smell of perspiration, and was quite warm: it was perfectly rigid.

"In this case, the aorta at its root, when cut open, measured three inches without being stretched, and was very thick at the same place; the pulmonary artery measured only two inches, and was quite natural.

"It may be naturally supposed that the inflammation of the œsophagus and stomach,—of the former especially, which was very decidedly marked,—should have produced some symptoms. The account which I obtained from a very close examination of the nurse who had attended him, was as follows:—That he was, in the first instance, admitted for bad legs; that he had been in the same ward for the same complaint some time before; and, in this last illness, had been a patient about a month; that, during the whole time, he had complained of a pain and a burning sensation about the pit of the stomach; that his appetite had been bad, and that during the last week he had scarcely ate any thing. He had had great difficulty in swallowing, and this action was accompanied with much distress. Two basins of sago was all he had taken for the last four days, and this with great difficulty. He had generally been very thirsty, and was fond of drinking cold water; nevertheless, his friends had secretly brought him wine and brandy, which he had also drank. On the Sunday night preceding his death, he had, after a trifling exertion, been exceedingly ill, and was almost insensible the whole night. On the Tuesday he was again taken the same way, and on the Wednesday he died. He had no stool for two or three days previous to his death, but, before that, had (according to the nurse's report) gone to the privy regularly.—This statement should be compared with the account of the appearance of the colon after death. There appears to have been no want of attention to the state of his bowels, as will be seen by the prescriptions which I subjoin. The case only shews that scybala will stand their ground against many purgatives. In the former part of his illness he had made rather a large quantity of urine: latterly it had been very scanty.

"The following are the prescriptions:—

"April 7. Inf. ros. cum magnes. sulph. (this was usually given at moderate intervals, in small doses, so as to produce gentle action of the bowels.)

"April 14. Elect. senn. comp. p. r. n.

"May 5. Sago cum vino; cerevisiæ ℔jſs. Inf. menth. sativ.

"May 6. Emp. lytt. capiti raso.

"Inf. sennæ cochleatim omni semi-hora donec, &c.

"Enema sapon. moll. si opus sit.

"That evening he died.

"Although so much disease of the vascular system was found after death, no very prominent symptoms existed during life; or, if they were present, they were confounded with those proceeding from the diseased state of the œsophagus and stomach.

"It is difficult to say what was the immediate cause of this man's death."

For ourselves, we are neither aware of any great quantity of disease in the vascular system, nor surprised at the man's death.

Three other cases follow; of which our limits will admit of no other notice than the author's concluding reflections.

"These two cases I have rather given on account of the singularity of the circumstances which attended them, than for any other reason: they go to prove what I have before stated, that where there is diminution of quantity, there is also diminution of cavity. For other examples of the same kind, in which, however, the calibre of the arteries was materially diminished, I shall, in a note, refer to other authorities,* as I am loth to encroach upon the time of the society by quoting cases, which, however, bear strongly on the point in question. Of those which I have taken the liberty of laying before it, the I. III. IV. VI. VIII. and IX. are probably deserving of attention; at least, there are peculiarities about them, which are by no means common; and they tend to illustrate some points of consequence in the pathology of these diseases.

"In maintaining the opinion, that alteration taking place in the minute vessels of the body (I mean the nutrient and secreting,) is a chief and principal cause of diseases of the heart, I do not pretend to discard the proper influence of the vital powers of that viscus itself in producing its own diseases. Indeed, from all the phenomena I observe, there appears to be very considerable power resident in it, of sympathising directly with other parts, which implies the property of receiving morbid impressions immediately; but, my reason for supposing that it seldom undergoes any morbid enlargement, independent of the effects produced upon it by the minute vessels of the body, is principally founded upon the fact, that the left ventricle is more commonly enlarged than any of the other cavities, which is precisely the one most likely to be affected by changes in these vessels; while the left auricle, which belongs to the same side of the heart, is more exempt from alteration of structure than any of the others; which is the reverse of what we might expect, if the morbid state were idiopathic. To develop, however, the different arguments which may be adduced in support of this opinion, and to compare them with those which might be placed on the opposite side, would occupy a great deal more time than I should feel justified in taking up at present; but I may, at some future period, endeavour to lay before the society the views I entertain on this particular subject more at large.

"* Vide Corvisart, Obs. XIX. p. 96.—Morgagni, Letter XVII. Art. 12."

"In detailing the cases, I have omitted to describe the palpitations, the sudden startings during sleep, the terrible anxiety, the coldness of the extremities, &c. &c. which are, almost invariably, concomitants of vascular disease. It would have taken up unnecessary time, since this is not a description of the malady, *but an enquiry into its nature and causes.* Also, I have, for the most part, omitted to say any thing of the treatment; and that for two reasons:—I wished to avoid doing so, as it was not directed by myself; secondly, they were cases in the last stage, which are unfortunately but too much beyond the reach of assistance, or, at any rate, of cure: but such it was necessary to select, as my object was to illustrate the nature of the symptoms during life, by the appearances after death.

"Lastly, I have said but little of the opinions which others have expressed on this subject. To have stated that Senac has warmly espoused the doctrine of obstruction in the minute vessels, would have rendered a long discussion necessary on the validity of his opinions, as supported by his arguments. It would have been out of place in the present instance. In speaking of solid œdema, I should not have omitted to mention the opinions of Dr. Blackall, Dr. Wills, and Dr. Grapengiassic, but that the cases here recorded, and the remarks made, were collected and noted previously to my having seen the observations of either. The remarks, indeed, under a somewhat different shape, were made the subject of an essay, which I read to the Medical Society of St. Bartholomew's Hospital, in the autumn of 1812, and was written in the spring and summer. The cases are almost altogether taken from among those I had observed before that period; and I have much to regret, that in consequence of not knowing at that time the coagulable nature of the fluid, separated from the vessels of the kidnies and other parts, in cases of this description, I could not make any observations on this point."

This "enquiry into the nature and causes," however laudable, we conceive a little premature in a student, and with cases, the early history of which, he had so few means of knowing. The paper is valuable, as recording dissections; but we suspect they were made immediately after a perusal of Corvissart, and some other French writers. We could very much wish, the English student would apply himself closer to our English physiologist. In perusing Mr. Hunter's account of the vascular system, he will meet with many remarks which will lessen his surprise concerning the size of the heart, and even the difference in its apparent bulk. When the body is stiffened after death, Mr. Hunter informs us, he has frequently found the heart in a relaxed state, and that, in this condition, it dilates according to the quantity of blood thrown into it. We presume, the *systematic* artery is the term used for the aorta, as the vessel from which the whole system is supplied. If this distinction, and change of name, were necessary, we should have preferred Mr. Hunter's, adopted by Bichât, of the *corporeal* and *pulmonary* circulation. Respecting aneurisms, we conceive our knowledge hitherto too imperfect to admit the term, either of active or passive. At present, we prefer names descriptive of appearance to those which would imply causes.

*Further Observations on the Ligature of Arteries; to which is added, a Case of Popliteal Aneurism, attended with some unusual Circumstances.* By WILLIAM LAWRENCE, Esq. F.R.S. Professor of Anatomy and Surgery to the Royal College of Surgeons, &c. &c.

Since the publication of Mr. Lawrence's former paper, he has found repeated causes in his own, and in the experience of others, to prefer his mode of tying arteries. We are not disposed to doubt his success; but still, we see so little reason to be dissatisfied with the old method of leaving the ends, that we feel not the least inclination to adopt this new plan. If any alteration is made, we could wish it should be without leaving any part of the ligature whatever; and, we very much suspect, that, in many cases, a short exposure of the artery, or a detachment from the surrounding parts, would have been sufficient. We have already proposed some experiments of this kind on criminals. They may, however, be tried with the utmost safety, if it were thought worth while, in all the operations for aneurism, according to the modern method. But, how little soever we may be disposed to adopt Mr. L.'s method, the subjoined case contains so many useful remarks, that we shall give it in the author's own words.

"A middle-aged man was received into St. Bartholomew's Hospital, with a large tumour filling up the whole ham, and extending on both sides of the femur towards the front of the limb. It had begun behind; had existed for five months; had grown latterly with great rapidity, and manifestly increased during a few days, of which we had the opportunity of observing it in the hospital. It had a firm fleshy feel, being a little softer at one of its anterior protuberances, than in other parts. It gave him great pain, though it was not tender on being handled: it had caused considerable œdema of the leg and foot, and had rendered the limb completely useless. The surgeons of the hospital, in consultation on this case, viewing it as a large and rapidly increasing fleshy tumour, determined that amputation of the limb was the only remedy that could be proposed. This I performed high up, having first plunged an abscess-lancet into the softest part of the tumour to the whole depth of the blade, without giving issue to any fluid.

"I employed pressure in the groin,* instead of the tourniquet; the use of that instrument being very unfavourable, where it is necessary to amputate in the middle of the thigh, and in a less degree in other amputations of this limb, by confining the muscles, and impeding their free retractions. This prevents us from sawing through the bone so high as we otherwise might do, and thus increases the chances of that very annoying occurrence, the protrusion and exfoliation of the bone.

"The examination of the amputated limb disclosed to us the very unexpected circumstance, that this tumour was a popliteal aneurism, containing an immense mass of firm bloody coagulum; not of that light brown laminated kind, which lines old aneurismal sacs, nor of the loose and soft texture that belongs to recently clotted blood. Hence, although the sac had been freely penetrated by the abscess-lancet, no part of its contents escaped.

"The coats of the popliteal artery, and a continuation of them, such as aneurisms ordinarily exhibit, formed the back part of the sac; while the front and sides were made up of the thigh-bone, the back of the knee-joint, and the neighbouring muscles. The fleshy and tendinous fibres of the vasti were exposed on clearing out the coagulum, which not only covered the back of the femur, but had also advanced on each side towards the front, so as nearly to have insulated the bone. The periosteum was removed at several points.

"The popliteal vein, stretched over the back of the tumour, was completely obliterated for some extent. The popliteal artery, similarly extended, was flattened for two or three inches, but quite pervious to the sac, as well as from it; both its openings into the bag presenting the usual appearances.

"When the patient was more closely questioned, after this examination of the limb, he stated that the swelling had continued of a moderate size until five weeks previous to his admission into the hospital, when it suddenly enlarged, and that it had increased con-

---

"* I had an instrument made for this purpose, which did not answer the end, in consequence of some inaccuracy in its construction; but, I conceive, that a similar one, properly formed, might advantageously supersede the tourniquet in amputations of the thigh. The instrument in question consists of a stout semicircle of iron, terminating behind in a flat circular piece, and perforated at its front-end by a screw connected to a small iron plate. It is placed on the pelvis like a truss. The posterior flat piece affords the counterpressure, and the screw in front will enable us to compress the artery to any degree that may be required. To the flat piece of iron, by which the artery is to be compressed, it will be necessary to fix a portion of cork, and cover it with leather. The circular part behind must also be suitably padded."

For the description of an instrument on the same principle, successfully employed for obliterating the humeral artery, without any cutting instrument; see our Journal, vol. vi. p. 537.—Edit.

siderably from that time. The pulsation, which the tumour no doubt had possessed at an early period, had altogether escaped his notice.

"I conclude, that the case had been originally a popliteal aneurism of the usual kind; that the sac had given way in front, so as to convert it from a circumscribed into a diffused aneurism, and thus to present to us the deceptive appearance of an immense sarcomatous tumour.*

"Since this case happened, I have heard of two or three other somewhat similar instances.

"The very large quantity of the coagulum, and the state of the thigh-bone, may create a doubt whether tying the femoral artery would have been a successful method of treating this case. However, had I suspected the nature of the affection, I should certainly have made the trial; and should have undertaken it with a confident expectation of success, grounded on experience of the efficacy and extent of those natural processes, by which such effusions are absorbed, and such cavities obliterated. I have stated the case to put others on their guard; and shall be happy, if what I have said should in any instance prevent so serious a mutilation as that which my patient suffered."

*A Case of Extra-uterine Fœtus, contained in the Fallopian Tube, with some Observations;* by Geo. Langstaff, Esq.

The public are already obliged to Mr. Langstaff for a very interesting description of an extra-uterine case in which the canal was obliterated, leading from that part of the tube, in which the ovum was found, to an uterus, without any *membrana decidua*. In the present case, the fallopian

"* An objection may be raised to this explanation, grounded on the general law of the animal economy, in [illegible] to which, aneurisms, as well as abscesses and tumours in [illegible], b[illegible], are observed to make their way toward the surface of the bessel a[illegible], in particular situations, towards the cavities of those mucous[illegible]s, which have external outlets. To this law there are, however, some apparent exceptions,—I say apparent, because the deviations may arise from some circumstance interfering with the ordinary progress of the tumours, such as a strain, effort, or external violence. I have dissected three or four thoracic and abdominal aneurisms, where the sacs had burst into the cellular substance; and I observed the same occurrence in one case of femoral aneurism. The instances in which aneurisms of the ascending or descending aorta burst respectively into the cavities of the pericardium or pleura, do not appear to be exceptions to the general rule; because the surrounding cellular substance, in both these cases, is not sufficiently copious to allow of the formation of a large swelling,—the sac, therefore, gives way when small, and must of necessity burst into the serous cavities just mentioned."

tube remained open, and the *decidua* was formed, the uterus somewhat enlarged; its mouth not closed, nor the mucous glands distended. The patient had menstruated regularly: death was occasioned by hæmorrhage into the cavity of the abdomen, in consequence of a rupture of the fallopian tube.

"On the left side, (says Mr. L.) the fimbriated part of the fallopian tube had formed adhesions to the posterior surface of the peritoneal covering of the uterus and the ovarium; so as to have completely precluded, during the person's life, the exact application of those parts, which is essential to generation."

"The above-mentioned appendages of the uterus were not brought into close union, but connected by very delicate longish layers of organised lymph, arranged in a reticulated manner."

Mr. Langstaff remarks, that he has seen similar appendages in women who have not proved prolific, and does not scruple to say, that such adhesions form the principal causes of barrenness. We confess ourselves not entirely satisfied with this reasoning, which, we conceive, would rather induce imperfect gestation than entire barrenness. However, as the author has paid great attention to the subject since he first entertained this doctrine, we conceive he must have confirmed it in so many instances as render it highly deserving the notice of every practical physiologist.

*The History of a Woman who bore a Seven Month's Fœtus, for Seven Years, was delivered of it per Anum, and completely recovered.* Communicated by Dr. ALBERS, of Bremen.

*On the Formation of New Joints;* by JOHN HOWSHIP, Esq. Member of [illegible] Royal College of Surgeons, &c.

This paper c[illegible]ins some further experiments and microscopical observ[illegible] of this accurate physiologist. We hope he will continu[illegible]eriments on inferior animals.

